# Neurotoxin (N-Oxalyl-L-α,β-Diamino Propionic Acid) Content in Different Plant Parts of Grass Pea (*Lathyrus sativus* L.) Spanning Seedling to Maturity Stage: Does It Increase over Time?

**DOI:** 10.3390/molecules27123683

**Published:** 2022-06-08

**Authors:** Surendra Barpete, Priyanka Gupta, Debjyoti Sen Gupta, Jitendra Kumar, Arpan Bhowmik, Shiv Kumar

**Affiliations:** 1 International Center for Agricultural Research in the Dry Areas (ICARDA), Rabat Institute, Rabat 6299, Morocco; vidhiguptaniwari@gmail.com; 2 ICARDA—Food Legume Research Platform, Amlaha 466113, India; 3 ICAR—Indian Institute of Pulses Research, Kanpur 208024, India; debgpb@gmail.com (D.S.G.); jitendra73@gmail.com (J.K.); 4 ICAR—Indian Agricultural Statistics Research Institute, Library Avenue, New Delhi 110012, India; arpan.stat@gmail.com

**Keywords:** ODAP, neurotoxin, grass pea, *Lathyrus sativus*, growth stage, neurolathyrism, non-protein amino acids

## Abstract

ODAP (N-oxalyl-L-2,3-diaminopropionic acid) is present in the seeds of grass pea. In this study, variation of total ODAP accumulation in leaves throughout the crop growth starting from 40 days after sowing to maturity, and the distribution pattern of ODAP in different plant parts including the seeds at the mature stage was analyzed. Five grass pea accessions were evaluated for two subsequent growing seasons in one location of ICARDA, Aleppo (Syria). The results found that the rate of accumulation of total ODAP varied during plant development. Increased rates of synthesis were noticed in young leaves of grass pea. The highest total ODAP content in leaves was noted in the early growth stage (40–50 days after sowing). Mean total ODAP content in leaves ranged from 0.17 to 0.96 percent during 2010–2011 and from 0.19 to 1.28 percent during 2011–2012. During maturity, the total ODAP content was lowest in the seeds than in leaves, stems, pod cover, seed coat, and cotyledons. The ranges of total ODAP content were 0.13 (seed)–0.34 (stem), 0.20 (seed)–1.01 (leaf), 0.22 (seed)–0.62 (leaf), 0.21 (seed)–0.66 (leaf), and 0.21 (seed)–0.78 (leaf) percent in B387, B222, B390, Bio-520, and B587 accessions, respectively, during maturity. The results indicated that the rate of accumulation and synthesis of total ODAP varied during the plant lifespan. The lowest total ODAP content of leaves was observed after 130 days of sowing. The lower total ODAP content after the early vegetative stage of grass pea plants makes them suitable as a feed.

## 1. Introduction

Grass pea (*Lathyrus sativus* L.) is considered an easy-to-adapt crop in a wide range of climatic conditions due to its demonstrated ability to survive under drought, intense precipitation events, elevated temperatures, and other edaphic stresses [1]. Its protein-rich seeds (>30% protein) are highly valued as human food and animal feeds. Grass pea assumes the role of survival food for poor masses and is the only source of feed and fodder for animals in rural areas of South Asia and Sub-Saharan Africa during drought years [2,3]. Continuous consumption of grass pea over long periods (>3 months) makes poor people vulnerable to ‘neurolathyrism’ due to a plant toxin called β-N-oxalyl-L-α, β-diaminopropionic acid (β-ODAP) present in its seeds and vegetative parts [4]. Lathyrism causes muscle spasms, cramps, and weakness of the lower limbs. Spastic paraparesis, sensory, and bladder dysfunction also may occur. Coarse tremor of upper parts was also observed. This medical condition is irreversible, however, mostly, life expectancy is not reduced [5]; Lambein et al. [6] postulated that since ODAP is an amino acid that accumulates in all tissues at all growth stages, it may have a role in drought tolerance that has made the grass pea such a useful species over long period of its domestication. However, homoarginine found in its seeds is an alternative substrate for the synthesis of Nitric Oxide (NO) in the human body that has a crucial role in the maintenance of cardiovascular and cerebral metabolism [7].

This crop remains popular among the resource-poor farmers in marginal areas due to the ease with which it can be grown successfully under adverse agro-climatic conditions without much production inputs [3,8,9], despite the ban on its sale in some countries [10]. Presently, it is grown on more than 1.5 million ha with 1.20 million tons production [1] and efforts are underway (in Australia, southern Europe, and America) to expand it as a break crop between cereals and as a bonus crop on fallow lands for feed and fodder [11,12]. However, the presence of ODAP in different plant parts of grass pea is the major hindrance in its further expansion as a food, feed, and fodder crop. Accumulation of ODAP content in grass pea cultivars is highly influenced by environmental factors [13,14,15]. An array of physiological and developmental processes influences ODAP content during ontogenetic changes in the life cycle of the plant under drought and heat stresses [1]. Therefore, due to the increasing interest in *Lathyrus* species as alternative pulses, grass pea breeding has a focus to reduce ODAP and increase methionine, homoarginine, and other nutritional factors [16,17,18]. Since grass pea plant is used as green fodder for animals and dry seeds for human food and animal feed purposes, the present study was undertaken to assess total ODAP concentration in developing plant parts at various growth stages to understand the pattern of total ODAP accumulation in grass pea. The objectives of this study were to find out (a) the total ODAP accumulation in leaf throughout the crop growth staring from 40 days after sowing to maturity, and (b) the distribution pattern of total ODAP in different plant parts, including seeds at the mature stage.

## 2. Materials and Methods

### 2.1. Plant Material

Five grass pea accessions (B222, B387, B390, B587, and Bio520) with similar phenology were selected for the present study in order to avoid the confounding effect of growth stages on the total ODAP content. The experiments were carried out at the International Center for Agricultural Research in the Dry Areas (ICARDA) at its experimental farm located at Tel Hadya (360°56′ E, 360°01′ N, 284 m AMSL) in Syria during 2010–2011 and 2011–2012. The soil at the experimental site is classified as clay with 7.9 pH and mean soil temperature ranging from 11.0 to 30.9 °C at 5 cm depth during the crop season. The climate of the region is typically the Mediterranean with hot dry summers and cold wet winters, with highly variable rainfall. The average precipitation during the crop season was 270 and 240 mm during 2010–2011 and 2011–2012, respectively. The experiments were carried out following the Randomized Complete Block Design (RCBD) with three replications. The individual plot size was 4.8 m^2^ with four rows of 4 m length spaced 30 cm apart. Seeds were sown at a 20 cm distance within the row for ease of sampling. No irrigation was given at any growth stage. Care was taken to ensure normal plant growth throughout the crop life cycle. 

### 2.2. Plant Material Sampling and Data Recording

To record leaf total ODAP concentration at different growth stages, plant samples were collected randomly from the middle rows of each plot at 10-day intervals, from 40 days after sowing until maturity. At the maturity stage, ten plants were harvested from each plot to obtain enough materials for the chemical analysis. During 2011–2012, reaching the maturity stage in addition to leaf samples different other plant parts were collected separately (shoot, pod cover, seed coat, cotyledons, and seed) for the total ODAP analysis. All the plant parts except the matured seeds were dried at 40 °C, weighed and carefully mixed to give reasonable representative samples for grinding. The matured seeds were air dried. The testa of seeds was not removed from any genotype during the study. 

### 2.3. Determination of Total ODAP Content

Total ODAP content in different plant parts and seeds was measured following UV-Spectrophotometer (Cadex Model: SB038, St-Jean-sur-Richelieu, QC, Canada) method suggested by Rao (1978) [19] and modified by Briggs et al., (1983) [20]. The modifications involved the two times extraction of 0.5 g of the flour with 60% ethanol followed by hydrolysis with 3M KOH (Sigma, St. Louis, MO, USA) in boiling water bath for 30 min. After centrifugation for 15 min, an aliquot (250 μL) of the hydrolysate was diluted with 750 μL water and reacted with 2 mL o-phthalaldehyde (Sigma, St. Louis, MO, USA) reagent. The mixture was incubated at 40 °C for 2 h before measuring the absorbance at 425 nm. The total ODAP standard curve (r^2^ = 0.99) was calibrated using DAP-HCl (Sigma, St. Louis, MO, USA).

### 2.4. Statistical Analysis

Repeated measures (RM)- analysis of variance (ANOVA) was performed on the mean-total ODAP content in leaf samples of five accessions for 2010–2011 and 2011–2012 using SAS (r) Proprietary Software 9.4 (SAS Institute, Cary, NC, USA). For each year, pairwise comparisons between all the level of time were done using *t* test. Here, *p*-value is adjusted based on Bonferroni Corrected Method. The effect size (*η*^2^) for RM-ANOVA which is measure of association between time and response variable was calculated as follows:

(1) $$\mathrm{Effect}\;\mathrm{Size}\left(\eta^{2}\right)=\frac{{\mathrm{SS}}_{\mathrm{Time}}}{\left({\mathrm{SS}}_{\mathrm{Time}}+{\mathrm{SS}}_{\mathrm{Error}}\right)}$$

Two-way analysis of variance was performed for total ODAP content among different plant parts in different lathyrus accessions during mature stage in 2011–2012. Equality of mean value of total ODAP content of tested accessions between growing years was calculated using *t* test. Similarly, equality of mean values of total ODAP content of specific growth stages between two growing years was also tested using *t* test. 

## 3. Results

### 3.1. Total ODAP Content in Tested Grass Pea Accessions in 2010–2011 

The highest total ODAP content was noted in early growth stage (40–50 days after sowing, DAS) (Table 1). The levels of total ODAP decreased apparently during later vegetative phase (>130 DAS), and subsequently remained lowest at mature stage in seeds of different accessions (Table 1). The ranges of distribution of total ODAP from early vegetative stage to maturity were 0.89 (40 DAS)–0.17% (maturity stage), 1.06 (50 DAS)–0.19% (maturity stage), 0.96 (40 DAS)–0.14% (maturity stage), 1.09 (50 DAS)–0.17 (maturity stage), and 1.03 (50 DAS)–0.21 (maturity stage) in accessions B387, B222, B390, Bio520, and B587, respectively (Table 1).

However, the genotype B390 showed lower total ODAP content compared to other four grass pea accessions at maturity stage. Repeated measures (RM)- analysis of variance (ANOVA) was calculated for total ODAP content in leaf samples of five accessions for 2010–2011 and found that time effect is significant at 1% level of significance (Table 2). The effect size (*η*^2^) for repeated measure ANOVA which is measure of association between time and response variable is calculated as follows:

(2) $$\mathrm{Effect}\;\mathrm{Size}\left(\eta^{2}\right)=\frac{{\mathrm{SS}}_{\mathrm{Time}}}{{(\mathrm{SS}}_{\mathrm{Time}}{+\mathrm{SS}}_{\mathrm{Error}})}=\frac{3.202}{3.429}=0.934$$

**Figure 1:**
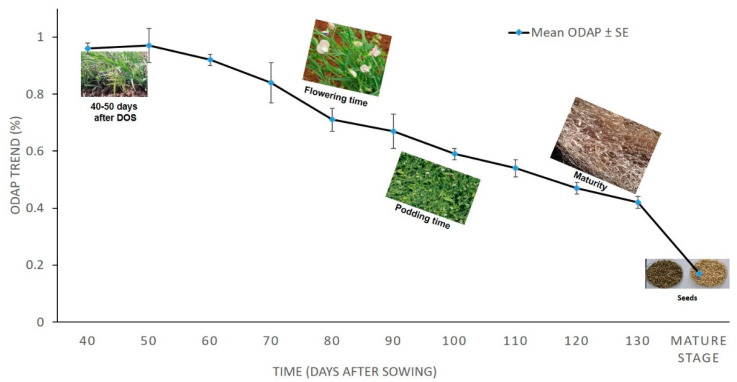
Mean leaf total ODAP content (%) of tested *Lathyrus* accessions grown during 2010–2011 at Tel Hadya. Y axis presents leaf total ODAP content in percent, X axis denotes Time, Time points were: 1 = 40 DAS, 2 = 50 DAS, 3 = 60 DAS, 4 = 70 DAS, 5 = 80 DAS, 6 = 90 DAS, 7 = 100 DAS, 8 = 110 DAS, 9 = 120 DAS, 10 = 130 DAS, and 11 = mature stage (seeds).

The effect size based on the above repeated measure ANOVA is 0.934, which is quite high enough and, thus, time (Days after sowing) is having significant impact on character (total ODAP content) under study. The trend in the total ODAP content accumulation in 2010–2011 as time increases has a downward movement (Figure 1). The mean neurotoxin content of five *Lathyrus* accessions from 40 DAS to maturity stage ranged from 0.97 to 0.17 percent (Table 3).

### 3.2. Total ODAP Content in Tested Grass Pea Accessions in 2011–2012

The highest total ODAP content was noted in the early growth stage (40 DAS) (Table 4). The levels of total ODAP decreased apparently during the later vegetative phase (>130 DAS), and subsequently remained lowest at the mature stage in seeds of all the accessions (Table 3). The ranges of distribution of total ODAP from early vegetative stage to maturity were 0.12 (maturity stage)–1.47 percent, 0.21 (maturity stage)–1.19 percent, 0.21 (maturity stage)–1.27 percent, 0.20 (maturity stage)–1.34 percent, and 0.20 (maturity stage)–1.13 percent in accessions B387, B222, B390, Bio520 and B587, respectively, at 40 DAS (Table 4).

However, the genotype B387 showed lower total ODAP content compared to the other four grass pea accessions at maturity stage (Table 4). Repeated measures (RM)—analysis of variance (ANOVA) was calculated for total ODAP content in leaf samples of five accessions for 2011–2012 and found that time effect is significant at 1% level of significance (Table 5). The effect size (*η*^2^) for repeated measure ANOVA which is measure of association between time and response variable is calculated as follows:

(3) $$\mathrm{Effect}\;\mathrm{Size}\left(\eta^{2}\right)=\frac{{\mathrm{SS}}_{\mathrm{Time}}}{{(\mathrm{SS}}_{\mathrm{Time}}{+\mathrm{SS}}_{\mathrm{Error}})}=\frac{4.152}{3.429}=0.908$$

**Figure 2:**
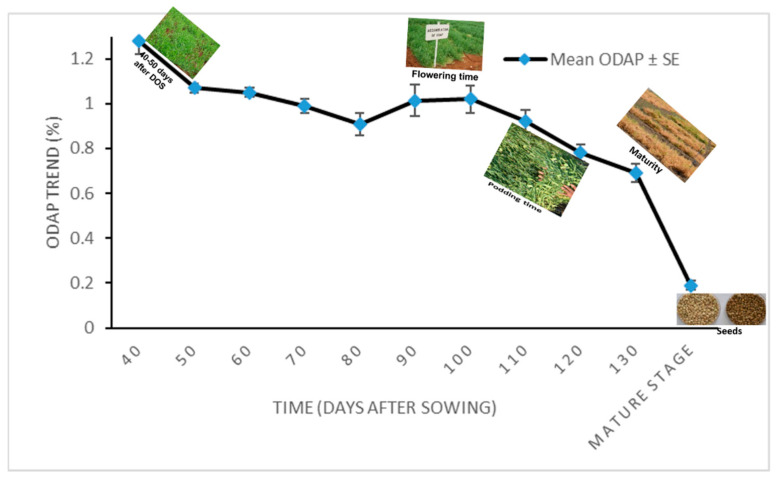
Mean leaf total ODAP content during different time points of sampling of tested *Lathyrus* accessions grown during 2011–2012 at Tel Hyada. Y axis presents leaf total ODAP content in percent, X axis denotes Time. Time points were: 1 = 40 DAS, 2 = 50 DAS, 3 = 60 DAS, 4 = 70 DAS, 5 = 80 DAS, 6 = 90 DAS, 7 = 100 DAS, 8 = 110 DAS, 9 = 120 DAS, 10 = 130 DAS, and 11 = mature stage (seeds).

The effect size based on the above repeated measure ANOVA is 0.908, which is quite high enough and, thus, time (Days after sowing) is having significant impact on character (total ODAP content) under study. The trend in the ODAP content accumulation in 2011–2012 as time increases has a downward movement (Figure 2). The mean of neurotoxin content for five *Lathyrus* accessions ranged from 1.28 to 0.19% from 40 days after seed germination to maturity stage, respectively (Table 6).

### 3.3. Distribution of Total ODAP in Different Plant Parts at Mature Stage

**Figure 3:**
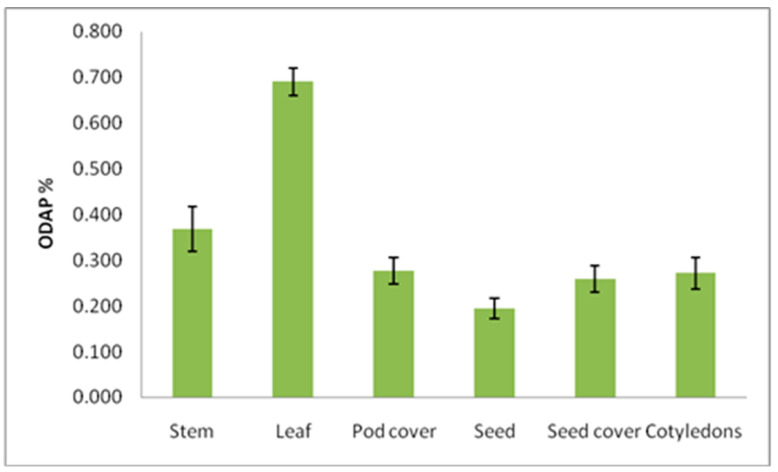
ODAP content variation in diffrent plant parts at mature stage in grass pea. Y axis presents ODAP content in percent, and X axis denotes different plant parts as source of sampling for ODAP estimation.

At the maturity stage when plants senescence, total ODAP content was determined in the stem, leaf, pod cover, seeds, seed coat, and cotyledons, and in ANOVA analysis, it was found that the tested genotypes significantly differed in total ODAP content (%) in case of leaf and seed coat samples. (Table 7 and Table 8, Figure 3). The total ODAP content was lowest in the seeds than in leaves, stems, pod cover, seed coat, and cotyledons (Figure 3). The ranges of total ODAP content were 0.13 (seed)–0.34 (stem), 0.20 (seed)–1.01 (leaf), 0.22 (seed)–0.62 (leaf), 0.21 (seed)–0.66 (leaf), and 0.21 (seed)–0.78 (leaf) percent in B387, B222, B390, Bio-520, and B587 accessions, respectively, during maturity. The leaves and stems seemed to be the major source of total ODAP (Table 8 and Figure 3) compared with different seed parts. The mean seed total ODAP content was lower compared to the mean total ODAP content of seed cover, pod cover, and cotyledons (Table 8 and Figure 3). The mean total ODAP content in pod cover, seed coat, and cotyledons were almost the same. All the accessions were having similar total ODAP content in seeds except for B387, which was having lowest total ODAP content (Table 8).

### 3.4. Yearly Variation in Total ODAP

The mean total ODAP content of *L. sativus* samples harvested during 2011–2012 was significantly higher (0.91%) (*t* test *p* value 0.042) compared to samples harvested during 2010–2011 (0.66%) (calculated from Table 6 and Table 3, respectively). The lowest seed total ODAP content (0.14%) was recorded in genotype B390 during the first growing season (2010–2011), and genotype B387 was having lowest seed total ODAP content (0.12%) during 2011–2012. While comparing the different plant stages between the two growing seasons (2010–2011 and 2011–2012), significant differences for total ODAP content (%) were observed for 40, 60, 80, 90, 100, 110, 120, and 130 days after sowing. Further, during maturity stage, no significant differences for total ODAP content (%) between the two growing years were observed (Table 9).

## 4. Discussion

### 4.1. Total ODAP Content in Tested Grass Pea Accessions in 2010–2011 and 2011–2012

The amount of total ODAP content at most developmental stages was significantly different except at maturity among all the accessions of grass pea between growth seasons 2010–2011 and 2011–2012 (Table 1, Table 4 and Table 9). During the early vegetative phase, rapid variation in the seed total ODAP content was noticed in all accessions. Moreover, total ODAP content in the seeds grown in field, showed similar trends during both seasons (Figure 1 and Figure 2). The data reported in this study were collected from two years of field experimentation, the pattern of accumulation and variation in the amount of total ODAP during different developmental stages varied during growth seasons as suggested by Addis & Narayan [21] and Jiao et al., [22]. However, it is not known whether the accumulation of total ODAP in tissues results from de novo synthesis in the tissue or translocation from other tissues.

The present results showed that most early developmental stages attained maximum total ODAP content and leaves are the most active site for total ODAP biosynthesis. It might be due to the increased level of free nitrogen-containing compounds in the cells during seed germination and early developmental stage [22]. Xiong et al., [15] reported that ODAP concentration varied significantly during vegetative and reproductive stages among the seven grass pea genotypes. The β-ODAP content decreased in leaves in early reproductive development and in pods as they matured. The net amount of β-ODAP in leaves and pods at early podding was positively associated with seed β-ODAP concentration at maturity. In the present study, a similar trend was also observed where total ODAP content gradually decreased from early vegetative phase to reproductive phase to maturity.

### 4.2. Changes in the Concentration of Total ODAP in Different Parts of Plants Depending on the Developmental Stage

The plants showed visible effects of ontogeny on total ODAP accumulation. Total ODAP content was low in the mature seeds compared to leaves, stems, pod cover, seed coat, and cotyledons (Table 7). Similarly, Xiong et al., [15] reported that ODAP content decreased in plant parts as they are matured. Kuo et al., [13] suggested that ODAP can be formed in the ripening seeds and is not exclusively transported from the ODAP pools in the pericarp. Jiao et al., [22] reported that total ODAP mainly accumulates in young or fast-growing tissues (including seedlings, ripening seeds, and young leaves), which are all sink tissues, containing a relative abundance of free nitrogen-containing compounds. In the present study, it was notified that during maturity, leaf of B222 exhibited higher (1.009%) total ODAP content compared to other accessions (Table 8). It might be due to the presence of high ODAP extracts, some of the tissues were colored with pigments. The previous report suggested that the presence of certain pigments and amino acids might affect the spectrophotometric measurement of total ODAP concentration [20,21]. On average, seed coat had 0.26% total ODAP (Table 7). However, pod cover on average showed a similar percentage (0.27%) of total ODAP. The mean total ODAP content in seed coat (0.26%) and pod cover (0.27%) agreed with the results of Addis and Narayan [21], who confirmed the rate of synthesis and accumulation of ODAP varied during the developing fruits of *L. sativus*.

### 4.3. Yearly Variation in Total ODAP

The level of total ODAP in harvested seeds of all accessions was substantially higher in the growing season 2011–2012 than 2010–2011 (Table 3 and Table 6). Piergiovanni et al. [23] reported that grass pea had widest year-to-year variation for seed total ODAP content as was recorded in the three *Lathyrus* accessions. Moreover, they also reported interesting considerations on factors affecting the ODAP content between locations as well as growing seasons for grain yield and ODAP content [23]. The ODAP storage in seeds changes from one year to the next, and the impact of growing location, might be relative to soil composition, yield, sowing and harvesting date, environmental conditions of growing location, genotype × environment interaction, etc. Similarly, as a consequence of the large overlap of sowing and harvesting periods between growing seasons, it is predictable that weather conditions (rainfall quantity and/or average temperature) might have a major influence in regulating the ODAP accumulation [24]. This is not surprising because it is well known that the amounts of some antinutritional factors (for example, ODAP and trypsin inhibitor content) stored in legume grains are modulated by high rainfall [23] and/or high temperature during specific crop growth stages [25,26]. The environmental conditions that can affect grass pea seed quality traits have been reported in recent studies dealing with morphological and compositional seed traits [23,27]. In the present study five (B222, 387, 390, Bio520, and 587) grass pea accessions introduced from ICARDA and were grown for December to June during 2010–2011 and 2011–2012 at the International Center for Agricultural Research in the Dry Areas, Aleppo, Syria, which is located at 360°56′ E, 360°01′ N latitude with an altitude of 284 m above sea level. The soil is clay with pH 7.9, with mean soil temperature in December to June ranging from 11.0 to 30.90 °C, respectively, at 5 cm depth. The weather variables during the testing periods for grass pea and average rainfall were 270 mm and 240 mm in 2010–2011 and 2011–2012, respectively. The lower rainfall could have caused higher mean total ODAP content of 2011–2012 grown *L. sativus* samples.

## Figures and Tables

**Table 1:** Total ODAP content (%) in different plant development stage in *L. sativus* during the year 2010–2011.

S. No.	Day *	Mean ± SE (%)				
		B387	B222	B390	Bio-520	B587
1	40	0.89 ± 0.11	1.02 ± 0.08	0.96 ± 0.10	0.93 ± 0.22	1.00 ± 0.15
2	50	0.75 ± 0.09	1.06 ± 0.10	0.94 ± 0.11	1.09 ± 0.20	1.03 ± 0.06
3	60	0.88 ± 0.20	0.93 ± 0.22	0.95 ± 0.07	0.89 ± 0.30	0.95 ± 0.28
4	70	0.70 ± 0.23	1.00 ± 0.25	0.87 ± 0.08	0.64 ± 0.21	0.99 ± 0.12
5	80	0.64 ± 0.18	0.80 ± 0.20	0.70 ± 0.15	0.59 ± 0.03	0.83 ± 0.07
6	90	0.70 ± 0.05	0.89 ± 0.09	0.59 ± 0.06	0.49 ± 0.20	0.66 ± 0.04
7	100	0.62 ± 0.20	0.66 ± 0.10	0.56 ± 0.22	0.53 ± 0.14	0.61 ± 0.09
8	110	0.59 ± 0.07	0.61 ± 0.05	0.49 ± 0.07	0.42 ± 0.17	0.57 ± 0.18
9	120	0.50 ± 0.03	0.54 ± 0.20	0.43 ± 0.23	0.39 ± 0.11	0.50 ± 0.09
10	130	0.48 ± 0.10	0.43 ± 0.20	0.39 ± 0.15	0.36 ± 0.15	0.43 ± 0.12
11	Mature stage (seeds)	0.17 ± 0.09	0.19 ± 0.10	0.14 ± 0.09	0.17 ± 0.10	0.21 ± 0.04

Day * = sample collection day after sowing.

**Table 2:** Repeated Measure ANOVA for leaf total ODAP content during 2010–2011.

Source of Variation	Df	Sum Sq	Mean Sq	F Value	Pr (>F)
Time	10	3.202	0.3202	56.43	<2 × 10^16^ *
Error (Genotype × Time)	40	0.227	0.0057		

* significant at 1% level of significance.

**Table 3:** Mean total ODAP content during different plant development stages in five accessions of grass pea during 2010–11.

* Days	40	50	60	70	80	90	100	110	120	130	Mature Stage (seeds)
** Mean ODAP% ± SE	0.96 ± 0.02	0.97 ± 0.06	0.92 ± 0.02	0.84 ± 0.07	0.71 ± 0.04	0.67 ± 0.06	0.59 ± 0.02	0.54 ± 0.03	0.47 ± 0.02	0.42 ± 0.02	0.17 ± 0.02

Day * = Sample collection day after sowing ** Mean total ODAP content in leaf of five acceccsions of grass pea.

**Table 4:** Total ODAP content (%) in different plant development stage in *L. sativus* during the year 2011–2012.

Sl. No	Day *	Mean ± SE (%)				
		B387	B222	B390	Bio-520	B587
1	40	1.47 ± 0.2	1.19 ± 0.06	1.27 ± 0.14	1.34 ± 0.19	1.13 ± 0.03
2	50	1.06 ± 0.04	1.02 ± 0.04	1.10 ± 0.08	1.10 ± 0.09	1.06 ± 0.07
3	60	1.04 ± 0.03	1.09 ± 0.04	1.01 ± 0.02	1.08 ± 0.05	1.03 ± 0.02
4	70	0.93 ± 0.06	0.94 ± 0.03	1.02 ± 0.02	1.10 ± 0.06	0.98 ± 0.02
5	80	0.80 ± 0.17	1.02 ± 0.24	0.81 ± 0.15	1.00 ± 0.20	0.91 ± 0.17
6	90	0.92 ± 0.21	1.08 ± 0.05	1.19 ± 0.10	1.37 ± 0.09	1.12 ± 0.08
7	100	1.22 ± 0.08	1.06 ± 0.11	0.86 ± 0.09	0.98 ± 0.16	0.98 ± 0.17
8	110	1.12 ± 0.27	0.92 ± 0.15	0.85 ± 0.12	0.89 ± 0.12	0.84 ± 0.14
9	120	0.74 ± 0.05	0.84 ± 0.13	0.76 ± 0.04	0.67 ± 0.02	0.90 ± 0.10
10	130	0.80 ± 0.20	0.58 ± 0.11	0.76 ± 0.06	0.74 ± 0.03	0.58 ± 0.10
11	Mature stage (seeds)	0.12 ± 0.03	0.21 ± 0.03	0.21 ± 0.04	0.20 ± 0.09	0.21 ± 0.22

Day * = sample collection day after sowing.

**Table 5:** Repeated Measure ANOVA for leaf total ODAP content during 2011–2012.

Source of Variation	Df	Sum Sq	Mean Sq	F Value	Pr (>F)
Time	10	4.152	0.4152	39.59	<2 × 10^16^ *
Error (Genotype × Time)	40	0.419	0.0105		

* Significant at 1% level of significance.

**Table 6:** Mean total ODAP content during different plant development stages in five accessions of grass pea during 2011–12.

Day *	40	50	60	70	80	90	100	110	120	130	Mature Stage (Seeds)
Mean ** ODAP% ± SE	1.28 ± 0.06	1.07 ± 0.02	1.05 ± 0.02	0.99 ± 0.03	0.91 ± 0.05	1.13 ± 0.07	1.02 ± 0.06	0.92 ± 0.05	0.78 ± 0.04	0.69 ± 0.04	0.19 ± 0.02

Day * = Sample collection day after sowing ** Mean total ODAP content in leaf of five acceccsions of grass pea.

**Table 7:** Analysis of Variance of Total ODAP content (%) of different *L. sativus* accessions at mature stage.

Source	DF	Mean Square (Stem)	Mean Square (Leaf)	Mean Square (Pod Cover)	Mean Square (Seed)	Mean Square (Seed Coat)	Mean Square (Cotyledons)
Rep	2	0.060	0.007	0.017	0.003	0.005	0.009
Genotypes	4	0.026	0.157 *	0.026	0.005	0.017 *	0.006
Error	8	0.032	0.019	0.017	0.001	0.004	0.003
Total	14						

* Significant at *p* < 0.05.

**Table 8:** Mean total ODAP content (%) in different plant parts at the mature stage in five *L. sativus* accessions grown during 2011–2012 at Tel Hyada, Aleppo, Syria.

Plant Part	Total ODAP Content (%) (Mean ± SE)					
	B387	B222	B390	Bio-520	B587	Total
Stem	0.342 ± 0.08	0.395 ± 0.02	0.266 ± 0.03	0.321 ± 0.03	0.514 ± 0.09	0.368 ± 0.05
Leaf	0.387 ± 0.05	1.009 ± 0.02	0.614 ± 0.06	0.663 ± 0.02	0.784 ± 0.01	0.691 ± 0.03
Pod cover	0.163 ± 0.01	0.285 ± 0.02	0.413 ± 0.08	0.219 ± 0.02	0.299 ± 0.02	0.276 ± 0.03
Seed	0.125 ± 0.02	0.200 ± 0.04	0.219 ± 0.04	0.211 ± 0.01	0.213 ± 0.03	0.193 ± 0.02
Seed coat	0.144 ± 0.03	0.260 ± 0.01	0.257 ± 0.06	0.354 ± 0.05	0.283 ± 0.03	0.260 ± 0.03
Cotyledons	0.181 ± 0.07	0.291 ± 0.02	0.249 ± 0.04	0.258 ± 0.02	0.379 ± 0.04	0.272 ± 0.04

**Table 9:** Mean total ODAP content (%) in different plant development stages in *L. sativus* during the year 2010–2011 and 2011–2012.

	Days of Sampling after Sowing										
Genotypes	40	50	60	70	80	90	100	110	120	130	Mature Seed
Mean	0.959 (2010) 1.278 (2011)	0.972 (2010) 1.066 (2011)	0.920 (2010) 1.048 (2011)	0.839 (2010) 0.992 (2011)	0.713 (2010) 0.907 (2011)	0.667 (2010) 1.134 (2011)	0.594 (2010) 1.021 (2011)	0.536 (2010) 0.922 (2011)	0.471 (2010) 0.782 (2011)	0.418 (2010) 0.692 (2011)	0.173 (2010) 0.193 (2011)
Variance	0.003 (2010) 0.018 (2011)	0.019 (2010) 0.001 (2011)	0.001 (2010) 0.001 (2011)	0.027 (2010) 0.005 (2011)	0.010 (2010) 0.011 (2011)	0.022 (2010) 0.027 (2011)	0.003 (2010) 0.017 (2011)	0.006 (2010) 0.013 (2011)	0.004 (2010) 0.008 (2011)	0.002 (2010) 0.010 (2011)	0.001 (2010) 0.002 (2011)
*t* test (*p* value)	0.004	0.173	0.000	0.107	0.017	0.002	0.001	0.000	0.000	0.002	0.364

Note: Number within parenthesis denotes year.

## Data Availability

The original contributions presented in the study are included in the article; further inquiries can be directed to the corresponding author/s.

## Abbreviations

ODAP	N-oxalyl-L-α, β-diaminopropionic acid
OPA	O-phthalaldehyde
DAS	Days after sowing
